# Supplementary figures and images for: Amplicon Sequence Variants Artificially Split Bacterial Genomes into Separate Clusters
Source: mSphere. 2021 Jul 21;6(4):e00191-21. doi: 10.1128/mSphere.00191-21 (PMC8386465; doi:10.1128/mSphere.00191-21)

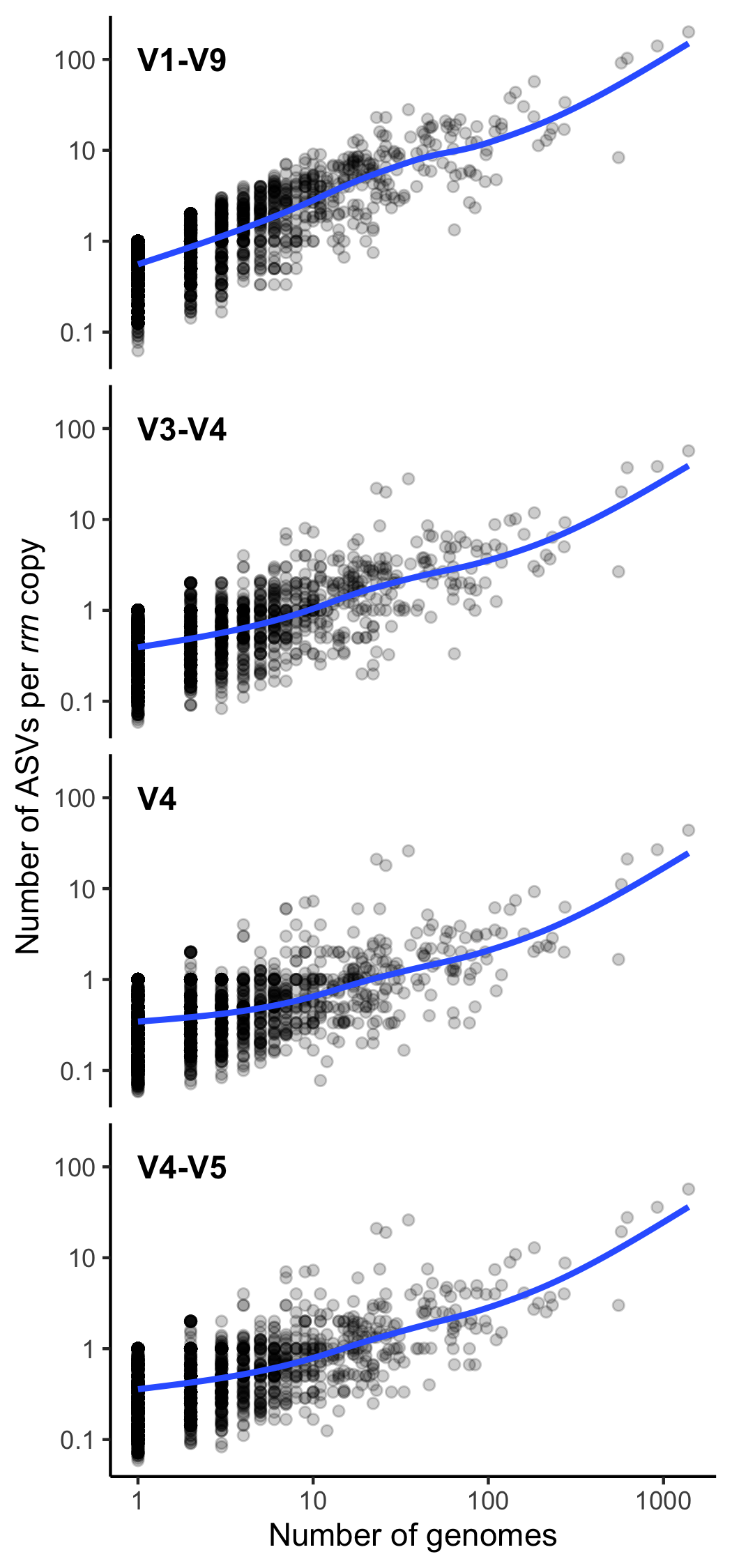

Supplement: FIG S1 [file msphere.00191-21-sf001.tif]
